# The practice-research nexus in allied health: practitioner-researchers or practitioners and researchers?

**DOI:** 10.1186/1757-1146-4-S1-P51

**Published:** 2011-05-20

**Authors:** Caroline J Robinson

**Affiliations:** 1 Charles Sturt University, Thurgoona, NSW, 2640, Australia

## 

*“I must admit before I did honours I thought you’d either be a researcher or you’d be a clinical podiatrist, but now that I’ve done both……well basically you need to be keeping up with your research so you know what to do clinically, so it’s all intertwined” *(Rachel)*.

How do you view yourself as an allied health professional - a clinical practitioner with no time for research, or a researcher spending little time in clinical practice? This presentation will reflect on the theme of ‘practice-research nexus’ which emerged from my PhD research, exploring the experience of allied health honours students. The traditional view of ‘research as enlightenment’ proposes that researchers generate novel ideas, test them, present them at conferences or publish them in journals, where they are available for the enlightenment of practitioners. ‘Research as retail’ suggests that research users want relevant, easy-to-understand evidence, summarised and placed conveniently for use as required. Both of these constructs are problematic and research-based practice infers a hierarchical relationship between theory and practice; research generates knowledge which determines practice. The honours student participants in this research were conscious of this ‘theory-practice gap’ and this was often highlighted by their experiences on clinical placement;

*“…..from what I’ve seen it’s easy to sit in your clinic room, close the door and not learn anything else……just come out of your degree and that’s it. One thing honours has taught me is that we don’t know everything yet….there’s so much more to learn” *(Ellie)*.

An increasing amount of readily accessible health and medical information places us as allied health practitioners in a position in which professional and public expectations are increased. We must be in a position to not only utilise research evidence in our practice, but also to contribute to health research and the generation of practice knowledge.

*“I have a better appreciation of research and how difficult it really is to do and I really believe in evidence based practice from doing honours. Why are we doing what we’re doing (in clinical practice) and why is that better than doing something else?” *(Nicola)*.

Through honours, students develop new perspectives on the relationship of research with clinical practice. There is a national imperative to reduce the degree of separation of research from clinical practice and honours is one of the mechanisms to develop the future practitioner-researchers, in allied health.

